# Lessons for Vietnam on the Use of Digital Technologies to Support Patient-Centered Care in Low- and Middle-Income Countries in the Asia-Pacific Region: Scoping Review

**DOI:** 10.2196/43224

**Published:** 2023-04-05

**Authors:** Leona Kosowicz, Kham Tran, Toan Tran Khanh, Thu Ha Dang, Van An Pham, Hue Ta Thi Kim, Hoang Thi Bach Duong, Tran Dong Nguyen, Anh Tuyet Phuong, Trong Hieu Le, Van Anh Ta, Nilmini Wickramasinghe, Penelope Schofield, John Zelcer, Tuan Pham Le, Tuan Anh Nguyen

**Affiliations:** 1 Social Gerontology Division National Ageing Research Institute Parkville Australia; 2 Department of Family Medicine Hanoi Medical University Hanoi Vietnam; 3 School of Health Sciences Swinburne University of Technology Melbourne Australia; 4 Hanoi Medical University Hanoi Vietnam; 5 New Horizon Palliative Care Company Limited Hanoi Vietnam; 6 Hanoi University of Science and Technology Hanoi Vietnam; 7 Hanoi University of Pharmacy Hanoi Vietnam; 8 Iverson Health Innovation Research Institute Swinburne University of Technology Melbourne Australia; 9 Military and Civil Medical Association of Vietnam Hanoi Vietnam; 10 UniSA Clinical and Health Sciences University of South Australia Adelaide Australia; 11 Health Strategy and Policy Institute Ministry of Health of Vietnam Hanoi Vietnam

**Keywords:** digital health technologies, digital health, eHealth, mobile health, mHealth, patient-centered care, Vietnam, Asia-Pacific region, digital, disease, technology, database, self-management, clinical, users

## Abstract

**Background:**

A rapidly aging population, a shifting disease burden and the ongoing threat of infectious disease outbreaks pose major concerns for Vietnam’s health care system. Health disparities are evident in many parts of the country, especially in rural areas, and the population faces inequitable access to patient-centered health care. Vietnam must therefore explore and implement advanced solutions to the provision of patient-centered care, with a view to reducing pressures on the health care system simultaneously. The use of digital health technologies (DHTs) may be one of these solutions.

**Objective:**

This study aimed to identify the application of DHTs to support the provision of patient-centered care in low- and middle-income countries in the Asia-Pacific region (APR) and to draw lessons for Vietnam.

**Methods:**

A scoping review was undertaken. Systematic searches of 7 databases were conducted in January 2022 to identify publications on DHTs and patient-centered care in the APR. Thematic analysis was conducted, and DHTs were classified using the National Institute for Health and Care Excellence evidence standards framework for DHTs (tiers A, B, and C). Reporting was in line with the PRISMA-ScR (Preferred Reporting Items for Systematic Reviews and Meta-Analyses extension for Scoping Reviews) guidelines.

**Results:**

Of the 264 publications identified, 45 (17%) met the inclusion criteria. The majority of the DHTs were classified as tier C (15/33, 45%), followed by tier B (14/33, 42%) and tier A (4/33, 12%). At an individual level, DHTs increased accessibility of health care and health-related information, supported individuals in self-management, and led to improvements in clinical and quality-of-life outcomes. At a systems level, DHTs supported patient-centered outcomes by increasing efficiency, reducing strain on health care resources, and supporting patient-centered clinical practice. The most frequently reported enablers for the use of DHTs for patient-centered care included alignment of DHTs with users’ individual needs, ease of use, availability of direct support from health care professionals, provision of technical support as well as user education and training, appropriate governance of privacy and security, and cross-sectorial collaboration. Common barriers included low user literacy and digital literacy, limited user access to DHT infrastructure, and a lack of policies and protocols to guide the implementation and use of DHTs.

**Conclusions:**

The use of DHTs is a viable option to increase equitable access to quality, patient-centered care across Vietnam and simultaneously reduce pressures on the health care system. Vietnam can take advantage of the lessons learned by other low- and middle-income countries in the APR when developing a national road map to digital health transformation. Recommendations that Vietnamese policy makers may consider include emphasizing stakeholder engagement, strengthening digital literacy, supporting the improvement of DHT infrastructure, increasing cross-sectorial collaboration, strengthening governance of cybersecurity, and leading the way in DHT uptake.

## Introduction

### Background

Vietnam’s health care landscape is changing. The country’s population is aging rapidly, with more than 1 in 5 Vietnamese citizens being predicted to be aged >65 years by 2050 [1]. It is forecast that Vietnam will transition from its current classification as an aging country, where 7% of the population is aged ≥65 years, to an aged country (ie, 14% of the population aged ≥65 years) in just 16 years [1]. By contrast, nearby countries Thailand and Singapore will take 20 years and 22 years, respectively, to reach this point [1]. This rapid aging is contributing to a shift in disease burden from communicable diseases to noncommunicable diseases (NCDs), that is, diseases that are not transmitted among persons but rather are the result of genetic, physiological, environmental, and behavioral factors [2].

In 2019, NCDs such as cardiovascular diseases, diabetes, and Alzheimer disease made up 8 of the top 10 causes of death in Vietnam for males and females across all age groups [3]. Furthermore, global health estimates published by the World Health Organization in 2020 showed that the percentage of deaths caused by NCDs in Vietnam has increased from 73% to 81% in <20 years [3]. This presents a major problem for the country’s health care system. NCDs are typically chronic and multimorbid and therefore require coordinated, long-term care [4]. Preventive measures for NCDs are also challenging, given the numerous risk factors associated with NCD onset [4]. Prevention and management of NCDs consequently demands considerable resources from all areas of the health care system.

By contrast, potential infectious disease outbreaks continue to threaten the health care system, and additional resources must remain on standby to cope with such eventualities. Vietnam’s existing health care system is not adequately resourced to meet these challenges. Health disparities are evident in many parts of the country, especially in rural areas, and the population faces inequitable access to quality, patient-centered health care [5]. This raises concerns since patient-centered care is widely considered to be an effective approach to health care from the perspective of patients, families, and health care professionals, and may also reduce health care costs [6-8]. Vietnam must therefore explore and implement advanced solutions to the provision of patient-centered care, with a view to reducing pressures on the health care system simultaneously.

The use of digital health technologies (DHTs) may be one of these solutions. Digital health refers to “the use of information and communications technologies in medicine and other health professions to manage illnesses and health risks and to promote wellness” [9]. This may include but is not limited to the use of wearable devices, mobile health (mHealth), telehealth, health IT, and telemedicine. DHTs have been shown to be effective in supporting the management of both NCDs, such as diabetes and cardiovascular disease, and infectious diseases, such as COVID-19 [10-17]. Evidence suggests that DHTs may also support several dimensions of patient-centered care, such as health knowledge, self-efficacy, quality of life, and access to health care [18,19].

### Objectives

Although there is increasing research demonstrating the value of DHTs in general, the potential of DHTs to support patient-centered care in Vietnam has thus far been relatively unexplored. Many neighboring low- and middle-income countries (LMICs) in the Asia-Pacific region (APR) are already exploring or implementing DHTs within their health care systems. This offers Vietnam the opportunity to gain insight into the effective use of DHTs from countries that share economic and cultural similarities, and to apply these learnings when developing its own approach to the use of DHTs to support patient-centered care for patients with communicable diseases and those with NCDs. This paper therefore aimed to identify the application of DHTs to support the provision of patient-centered care in LMICs in the APR and to draw lessons for Vietnam.

## Methods

### Study Design

A scoping review protocol was developed and registered on the Open Science Framework [20]. The review was undertaken using the following established methodologies: (1) identifying the research questions; (2) identifying relevant studies; (3) study selection; (4) charting the data; and (5) collating, summarizing, and reporting the results [21,22]. Reporting was in line with the PRISMA-ScR (Preferred Reporting Items for Systematic Reviews and Meta-Analyses extension for Scoping Reviews) guidelines (Multimedia Appendix 1 [21-23]).

### Review Questions

The research questions guiding this review were as follows:

1. What types of DHTs are being used in LMICs in the APR?
2. What patient-centered outcomes are associated with the use of DHTs?
3. What are the enablers and barriers for the use of DHTs to support patient-centered care outcomes?
4. What lessons can Vietnam learn when developing its own approach to the use of DHTs to support patient-centered care?

### Search Strategy

Using search terms related to DHTs and patient-centered care, we conducted a comprehensive search in January 2022 in the following 8 electronic databases: MEDLINE, PubMed, Embase, EMCare, PsycInfo, Ovid Nursing Database, Web of Science, and Scopus. The search strategy for MEDLINE is presented in Multimedia Appendix 2.

Studies were considered eligible if they met the following criteria: (1) published in English or Vietnamese; (2) set in LMICs in the APR; (3) discussed communicable diseases or NCDs; and (4) discussed the application of DHTs to support patient-centered care with regard to patient-centered outcomes, barriers and enablers for the use of DHTs, and policy or practice outcomes. LMICs were defined according to the relevant 2022 World Bank country classifications: low-income economies (gross national income of ≤US $1085 per capita), lower middle-income economies (gross national income between US $1086 and US $4255 per capita), and upper middle-income economies (gross national income between US $4256 and US $13,205 per capita) [24]. When selecting LMICs for inclusion, we applied the World Bank definition of the APR, which includes countries geographically neighboring Vietnam within East Asia and the Pacific [24]. The search was not limited by publication date or type, although publications that did not present outcomes (eg, protocol papers) were excluded at the screening stage.

The database searches were supplemented by manual searches and references as appropriate. Duplicates were removed using an EndNote library (Clarivate) [25], and the remaining titles were imported into Covidence software (Veritas Health Innovation Ltd) for screening [26].

### Study Selection

Interrater reliability of the screening process was established using an initial selection of 5 publications that were independently screened at the title and abstract and full-text levels by 4 reviewers. Discrepancies in screening decisions were discussed and resolved by consensus before the final inclusion and exclusion criteria were agreed upon. The titles and abstracts of the remaining publications were then independently screened by 2 reviewers per publication before 2 reviewers completed a full-text review of each publication remaining thereafter. Conflicts were resolved through consensus.

### Data Extraction

A data extraction form was developed to identify the key characteristics of each study as well as relevant information regarding the application of DHTs in the provision of patient-centered care. Seven reviewers independently extracted the data and resolved inconsistencies through discussion with 2 additional researchers. The variables included authors, publication year, country of origin, aims, settings, study design, methodology, type of DHT, reported outcomes, enablers, barriers, and policy and practice implications.

### Data Synthesis

Thematic analysis was used to synthesize and report the findings, following the approach described by Braun and Clarke [27]. This involved (1) familiarization with the data, (2) searching for themes, (3) reviewing the themes, (4) defining and naming the themes, and (5) producing the report.

Outcomes were considered patient centered if they mapped against established definitions and determinants of patient-centered care, that is, health care that aligns with patients’ values, needs, and preferences, as well as increases patient autonomy and involvement in their care [28]. Systems-level determinants of patient-centered care were also considered in addition to this definition, including factors related to system characteristics and structures and processes, as well as external policies, regulations, and resources [29].

Further to the thematic analysis, the full texts of the selected articles were analyzed to identify the types of DHTs used. The DHTs were then grouped according to classifications set out by the National Institute for Health and Care Excellence evidence standards framework for DHTs [30]. This framework classifies DHTs by intended purpose and stratifies them into 3 tiers based on the potential risk to service users and to the system (tier A, tier B, and tier C). Tier A comprises DHTs intended to save costs, release staff time, or improve efficiency; tier B includes DHTs that help citizens and patients to manage their own health and wellness; and tier C comprises DHTs used for treating and diagnosing medical conditions or for guiding care choices [30]. Each tier is further divided into subcategories that relate to the intended purpose of the DHT in question.

### Ethical Considerations

Ethics approval was not required for this review paper.

## Results

### Overview

**Figure 1:**
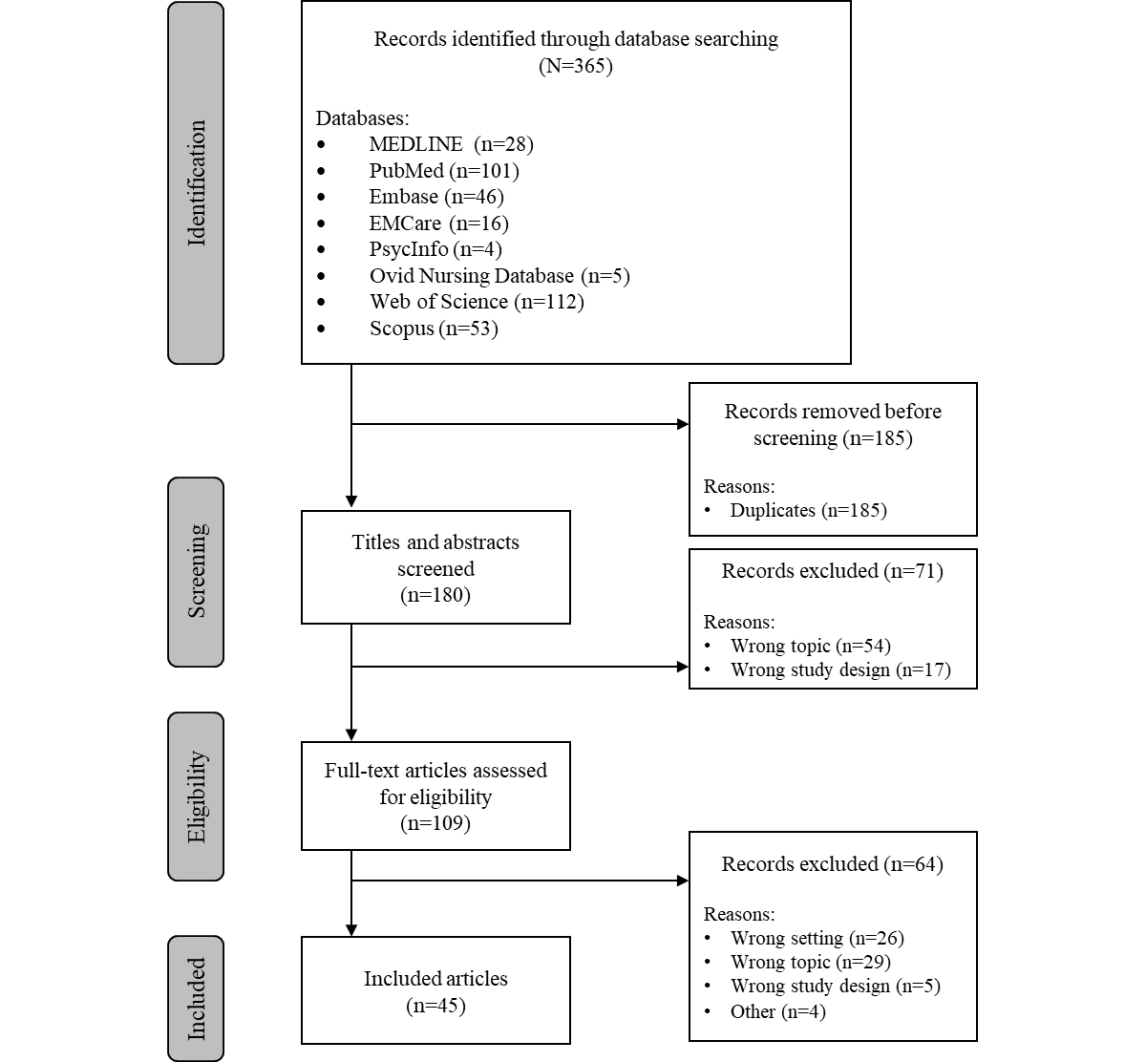
PRISMA (Preferred Reporting Items for Systematic Reviews and Meta-Analyses) diagram of the study selection process.

A total of 264 publications were identified through the database search, of which 45 (17%) were included in the final analysis (Figure 1). Of these 45 articles, 19 (42%) were quantitative studies (including n=3, 16%, randomized controlled trials), 7 (16%) were qualitative studies, 4 (9%) were mixed methods studies, 6 (13%) were technical reports, and 9 (20%) were review papers. The included articles were published between 2010 and 2021, with a majority published in 2020 (11/45, 24%) and 2021 (13/45, 29%). Most of the studies were conducted in India (14/45, 31%) and China (11/45,24%). Studies from Malaysia, Pakistan, Bangladesh, Indonesia, Nepal, Sri Lanka, Thailand, and Vietnam were also included. Of the 45 publications, 20 (44%) focused on NCDs (eg, diabetes, cardiovascular disease, and mental health conditions), 3 (7%) focused on communicable diseases (eg, COVID-19, tuberculosis, and acute diarrhea), and 5 (11%) encompassed both communicable diseases and NCDs, whereas the remaining publications (17/45, 38%) did not focus on a specific health condition or did not include this information. Of the 45 publications, 33 (73%) presented data on novel or specific DHTs, whereas the remaining studies (12/45, 27%) mapped the existing landscape of DHT-supported health care in specific countries or populations or provided evidence on enablers and barriers for DHT-supported health care. Detailed characteristics of the included studies are presented in Multimedia Appendix 3 [30-75].

### Classification of DHTs

Among the 33 studies presenting novel or specific DHTs, the majority of the DHTs were classified as tier C (n=15, 45%), followed by tier B (n=14, 42%) and tier A (n=4, 12%). The DHTs identified were further classified by intended purpose (Textbox 1 [30]).

**Summary of identified digital health technologies classified according to the National Institute for Health and Care Excellence evidence standards framework for digital health and care technologies.**

Tier A

- System services: health information systems [31,32] and mobile apps (medical document digitizer [33] and patient appointment flow optimizer [34])

Tier B

- Communicating about health and care: telemedicine and teleconsultation platforms (telephone, SMS text messaging, and video) [35-42] and appointment reminders [36]
- Health and care diaries: mobile apps to track and record users’ health information for self-monitoring [43,44]
- Promoting good health: internet-based health information [45,46] and nonpersonalized health education via SMS text messaging [47,48]

Tier C

- Inform clinical management: mobile apps to allow remote monitoring of patient health information to provide personalized recommendations direct to users and inform clinical decision-making by health care professionals [49-59]
- Diagnose a condition: artificial intelligence–based self-diagnosis tool [60]; mobile-based assessment tools [61,62]; and software solution to allow mobile monitoring, assessment, and diagnosis [63]

### Patient-Centered Outcomes of DHTs

In studies that evaluated the outcomes of DHT use, DHTs supported patient-centered outcomes at an individual level and a systems level.

#### Individual-Level Outcomes

At an individual level, DHTs increased accessibility of health care and health-related information, supported individuals to self-manage their health, and led to improvements in clinical and quality-of-life outcomes.

##### Increased Accessibility

Nedungadi et al [58] designed and pilot-tested a self-monitoring system for managing well-being in rural areas of India. The device was shown to accurately monitor patient conditions, was easy to understand and valued by patients, and was able to be delivered at a low cost. Overall, the device contributed to filling a gap in access to health care and health-related information in rural Indian locations, where health care resources are scarce. An mHealth device for diabetes management and education developed in China showed similar results [64]. The multimedia teaching platform within the device enabled users to easily access diabetes-related information, which was identified as an unmet need within the diabetes community. Secondary benefits of DHTs that increased accessibility included reduced travel times and reduced health care–related costs for both patients and their families [42,62,65]. The findings about increased accessibility and the associated benefits were further confirmed in qualitative studies that explored the benefits of mHealth and internet hospitals [37,66], as well as in review papers that addressed this topic [65,67,68]. Overall, these DHTs promoted patient-centered outcomes by providing flexible health care options that met patients’ individual access needs.

##### Improved Self-management

DHTs were shown to support patients in self-monitoring and self-managing diabetes, COVID-19 infection, medication adherence, cardiovascular disease, and general health, increasing patients’ involvement in their own health care [43,50,53,58,64,69]; for example, Vitale et al [53] developed a diabetes telemanagement system that was found to improve diabetes self-management in terms of frequency of blood glucose monitoring and frequency of insulin use. A COVID-19 symptom monitoring system developed by Lim et al [50] supported patient decision-making with regard to symptom severity and actions required. Finally, Chew et al [43] found evidence of improved medication adherence in people taking long-term medications who used a novel medication adherence app. A systematic review that explored the use of DHTs for self-management of cardiovascular disease reported that mHealth platforms can improve patient knowledge and confidence in self-management, increase active symptom monitoring and recording, and improve adherence to medications and appointments [69].

##### Improved Clinical and Quality-of-Life Outcomes

In a cluster randomized controlled trial, Guo et al [49] compared the use of an mHealth platform to usual care for the management of patients with atrial fibrillation. Patients in the intervention group had significantly lower rates of the composite outcome “ischemic stroke/systemic thromboembolism, death, and rehospitalization” (*P*<.001) as well as consistently lower heart rates than those receiving usual care. In a cross-sectional study by Vitale et al [53], patients with diabetes who received comprehensive care involving teleconsultations achieved better intermediate health outcomes than those receiving usual care (ie, significantly lower glycated hemoglobin: *P*=.003, cholesterol: *P*<.001, and diastolic blood pressure: *P*=.02). Finally, a WeChat-based intervention implemented in China reduced depressive symptoms in participants who participated for a 3-month period [55]. In contrast to these studies, a randomized controlled trial assessing the effectiveness of an SMS text messaging system for managing coronary heart disease reported no significant changes in any clinical outcomes measured [48]. A single study specifically reported on quality-of-life outcomes related to DHT use. Gupta et al [62] developed and implemented a telemedicine device for otology screening in rural India. Use of the device resulted in 265,615 referrals, and 45% (9443/20,986) of the referred patients who reported for and received treatment reported a “significant improvement in their quality of life.” Finally, in an evaluation of a mobile obstetrics monitoring platform, patients who received care using the platform almost unanimously reported an increased feeling of safety while being remotely monitored, which may be considered an aspect of quality of life [63]. On the whole, these DHTs contributed to patient-centered outcomes by enabling access to health care that was effective and met patients’ clinical and psychosocial needs.

#### Systems-Level Outcomes

DHTs also supported systems-level determinants of patient-centered outcomes, including increased efficiency, reduced strain on health care resources, and support for patient-centered clinical practice.

##### Increased Efficiency

In some of the studies (6/45, 13%), DHTs reduced the amount of time clinical staff spent undertaking administrative tasks and thereby increased their availability for tasks that directly benefited patients. Ali et al [33] trialed a mobile app for document digitization in hospitals and found a considerable time reduction in data aggregation and data transfer activities. Similarly, an eHealth system implemented at a primary health care center in India reduced the amount of time that staff spent generating reports [31]. Another group of researchers developed a mobile app to improve patient flow during hospital appointments [34]. The app was shown to reduce the number of times patients requested appointment information from hospital staff and to reduce the amount of time staff spent seeking appointment-related information and responding to patients. A qualitative study reported that mHealth allows staff to have timely access to patient records at the time of treatment [66], and this finding was echoed in the evaluation of the mobile obstetrics monitoring platform described earlier, which allowed health care workers to remotely view patient records [63]. A narrative review paper also highlighted the role of DHTs in increasing operational efficiencies [67].

##### Reduced Strain on Health Care Resources

DHTs supported reducing strain on health care resources in 3 ways. First, DHTs were shown to facilitate remote monitoring of patients’ health status [39,50,66,69]; for example, the COVID-19 symptom monitoring system designed by Lim et al [50] monitored patient stability, connected health care professionals and patients via teleconsultations, and alerted patients and health care professionals to changes in, and worsening of, symptoms. Most of the patients were thereby enabled to recover at home rather than needing to be hospitalized, reducing unnecessary use of health care resources and simultaneously supporting patient preference. Reductions in emergency conditions and resultant hospitalizations also emerged as benefits of remote monitoring in several other publications [49,58,69]. Second, DHTs reduced the need for referral to other health care professionals or diagnostic services. According to a narrative review, the use of teleconsultations coupled with services such as teleradiology and telepathology may enable patients to receive advice and diagnoses in a shorter time period and without the need for referral to specialists, thereby freeing up specialist availability while also providing patients with faster access to the care they need [65]. Third and last, DHTs enabled patients to be triaged to a care mode that best suited their needs and preferences [38,50,62,65,70]. As in the case of remote monitoring, this enabled some patients to receive remote care rather than receiving face-to-face care and thereby contributed to both meeting patient preferences and the conservation of clinic and hospital resources.

##### Support for Patient-Centered Clinical Practice

In 9% (4/45) of the studies, DHTs were reported to support health care staff in efficient decision-making, accurate assessment, and timely diagnosis [31,36,61,63]. This enabled health care staff to provide care that was closely aligned with patients’ clinical needs, whereby patients received the right care at the right time. DHTs also improved continuity of care (eg, by reducing repeated patient and health care provider interactions) [50,66] and facilitated multidisciplinary teamwork by connecting different health care professionals remotely [63]. For patients, this translated into a more seamless health care journey and enabled them to receive holistic health care from a number of disciplines when required.

### Enablers and Barriers for the Use of DHTs for Patient-Centered Care

Enablers and barriers for the use of DHTs for patient-centered care emerged at the level of the device or platform, the user, and the broader environment (Table 1). At the device or platform level (ie, characteristics and design of DHTs), the most commonly reported enablers related to the ability of DHTs to meet users’ individual needs, such as those that integrated easily with users’ lives or workflows [33,40,52,57,64,69] and those that were adapted to local languages, cultures, and literacy levels [32,46,54,58,64,71]. Co-design methodology was commonly suggested as an enabler to developing DHTs that aligned with users’ individual needs [40,43,50,51,56,59,67]. Incorporation of direct support from health care professionals (eg, teleconsultations) was also recommended [35,40,47,50,64,69], as was ensuring that users found DHTs easy to use [52,54,60,66,69,72]. Although no barriers to patient-centered outcomes pertaining to the characteristics or design of DHTs were specifically reported in these studies, each of the enablers, if considered in reverse, could be considered barriers (eg, DHTs that are not adapted to local languages).

Regarding the user level, the availability of technical support and user education and training with regard to DHTs was the most commonly reported enabler [31,41,50,54,60,62,65,66,68], whereas low literacy and technical literacy emerged as a user-level barrier in several of the publications (9/45, 20%) [38,40,44,46,47,54,65,66,69].

**Table 1:** Enablers and barriers for the use of digital health technologies (DHTs) for patient-centered care.

	Enablers	Barriers
Device or platform level	Uses triaging to assess users’ suitability for DHT-supported vs face-to-face care [38,50,64] DHT is individualized to users’ needs [45,46,55,66,71] Platform type suits the needs of the user [38,46,65] Allows personalized monitoring and self-management [57,58,67,69] DHT use integrates with users’ daily lives and workflows [33,40,52,57,64,69] Adapted to local language, culture, context, and literacy levels [32,46,54,58,64,71] Provides accurate and clear information [40,54,60] Incorporates direct support from health care professionals [35,40,47,50,64,69] Content is adaptable to changing clinical evidence [50,54] Enables multidisciplinary collaboration [63] Uses centralized, sharable data [32,64,66,67] Validated for use in the proposed context [39] Co-designed in collaboration with end users (eg, considers their needs and concerns) [40,43,50,51,56,59,67] DHTs that are easy and fun to use [52,54,60,66,69,72] Adequate device performance (eg, battery life and processing speed) [54,58] Data stored, shared, and used securely [42,66]	None specifically reported
User level	Adequate provider-patient relationship building [38,41,66] Technical support, training, or education available for users [31,41,50,54,60,62,65,66,68] User willingness to adapt and positive attitude toward change [68] Behavioral factors such as perception of DHTs as trustworthy [41,54,68], useful [68,72], and able to meet users’ needs and health goals [52,72]	Low literacy and technology literacy [38,40,44,46,47,54,65,66,69] User concerns around the following aspects: Privacy and security [51,67-69] Financial risks [51] Self-ability to use DHTs [38,51,69] Effort, time consumption, and workload disruption [40,72] Credibility and reliability of content [44,69] Possible inaccuracy of user data entry or reporting [66] Difficulty developing patient-provider rapport via DHTs [38]
Environmental level	Decreasing prices of electronic devices in general [58,75] Improved mobile technology infrastructure [75] Community “hubs” that enable wider access to DHTs [65] Adequate DHT infrastructure (eg, in rural areas) [38,68,71,75] Positive promotion of DHTs to users [37,40,66,67] Governance that ensures security, privacy, and integrity of DHTs [32,65-67,71,73,74] Inclusion of DHT-based care in health insurance [37,71] Cross-sectorial collaboration on development, implementation, and promotion of DHTs [62,64,66,67,71]	Limited user access to mobile phones, computers, internet, and reliable electricity in some locations [31,38,46,65] High cost of implementing or accessing some DHTs [67,75] Environmental factors that limit users’ ability to independently implement lifestyle recommendations (eg, lack of public exercise spaces) [36,47] Lack of policies and protocols to guide DHT implementation and use [33,38,46,64,68]

Finally, at a broader environmental level, governance that ensures security, privacy, and integrity of DHTs [32,65-67,71,73,74] as well as cross-sectorial collaboration between and within government and nongovernment sectors on development, implementation, and promotion of DHTs [62,64,66,67,71] were the most frequently reported enablers. Limited user access to DHT infrastructure (especially among populations residing in rural areas and those with low socioeconomic status) [31,38,46,65] and a lack of policies and protocols to guide the implementation and use of DHTs [33,38,46,64,68] emerged as common barriers at the environmental level.

## Discussion

### Principal Findings

This scoping review is the first to bring together evidence regarding the use of DHTs to support patient-centered care in LMICs in the APR, contributing to an increase in the knowledge base about the value of DHTs in non-Western countries. The findings suggest that many LMICs in the APR are successfully using DHTs to support the equitable provision of patient-centered care and simultaneously reduce pressures on their health care systems. To optimize success when developing its own approach to the use of DHTs to address the country’s specific health care challenges, Vietnam should take advantage of the lessons learned by these neighboring countries. In line with the findings of previous studies, DHTs were shown to be a viable option for the management of NCDs such as diabetes, cardiovascular disease, and depression in LMICs in the APR, which is encouraging, given the rise of NCDs in Vietnam. Evidence from a study on remote monitoring of patients with COVID-19 infection also demonstrated the potential of DHTs to conserve health care resources in the face of communicable disease outbreaks [50]. Perhaps most promisingly, DHTs were able to increase access to health-related information and health care services in rural and low-income areas. This suggests that DHTs may go some way toward addressing the health disparities that persist in Vietnam.

However, both the development and implementation of DHTs to support patient-centered care were not without challenges. Although several enablers for the use of DHTs were identified, so too were many barriers at the user and environmental levels. These barriers need to be considered and accounted for when planning for widespread use of DHTs. It is also important to note that, although no barriers pertaining to the individual characteristics or design of DHTs were specifically reported, a lack of evidence regarding device- and platform-level barriers in the included studies does not indicate the absence of barriers at this level. Each of the enablers reported at the device and platform levels may also act as a barrier to patient-centered outcomes if not fulfilled. The same is true of enablers at the user and environmental levels. Therefore, policy makers should take a holistic view with regard to enablers and barriers and give equal weight to both when planning for DHT use. There are several recommendations that Vietnamese policy makers may consider.

First, it is important to emphasize stakeholder engagement. According to our findings, DHTs that strongly align with the needs of end users reflect patient-centered principles and are likely to enable patient-centered outcomes. Therefore, it is crucial to engage with end users at all stages of DHT development and implementation to understand and meet their needs. This could be achieved by adopting a co-design approach. Co-design has been well established as a methodology for the development and ongoing improvement of health care services [76]. It is defined as “collective creativity as it is applied across the whole span of a design process” [77], which in this context refers to the involvement of a diverse range of stakeholders (ie, patients and their carers, health care professionals, researchers, and technology designers) throughout the development and implementation of DHTs. Several studies identified in this review used elements of co-design to determine the feasibility, acceptability, and usability of DHTs, and this approach was strongly recommended to increase the likelihood of patient-centered outcomes [43,50,56,59].

Second, measures are needed to strengthen digital literacy among the Vietnamese population. According to the *Global Competitiveness Index 4.0 2019 Rankings*, which measure digital skills among the active population on a scale ranging from 1 to 7, Vietnam achieved a value of 3.8 [78]. Although Vietnam is ranked fourth in a list of 8 Association of Southeast Asian Nations member states in terms of digital literacy [79], it comes in 97th when compared with global estimates among 141 countries [78]. Our findings demonstrated that low digital literacy is a key barrier to the uptake of DHTs for both patients and health care staff and as such may restrict access to patient-centered health care if not addressed. Digital literacy education is therefore required. A multimodal approach is needed, including school-based education, community education and adult learning, and workforce training and development. This would enable the Vietnamese population to develop strong foundations in digital literacy, as well as improve their digital literacy in later life. For guidance, Vietnam may look to the World Health Organization’s Global Strategy on Digital Health 2020-2025, which includes improved digital literacy in its strategic objectives [80]. Our findings suggest that, even with adequate digital literacy, users value the availability of technical support and training for the use of specific DHTs. As such, DHT developers should consider this an essential component of their product.

Third, support is needed for the improvement of DHT infrastructure. Population-wide access to affordable and reliable mobile devices, computers, internet and mobile networks, and electricity is essential for the use of DHTs according to the studies included in this review. Promisingly, the Vietnamese government has already committed to improving the nation’s digital infrastructure in its National Digital Transformation Program 2025-2030 [81]. Targets include improvements to internet and mobile networks throughout the country, as well as the establishment of a telemedicine unit in 100% of the health care services. However, no measures or targets relate specifically to improving digital infrastructure in rural and low-income regions of Vietnam. Our findings indicate that these regions may require additional support to achieve equitable access to DHTs, using measures such as subsidized access to these technologies and inclusion of DHT-supported care in medical insurance plans [36-38,71].

Fourth, our findings highlight cross-sectorial collaboration on development, implementation, and promotion of DHTs as a key enabler for the use of DHTs to support patient-centered care. This aligns with findings from previous studies [82,83]. A coordinated, whole-system approach is needed to overcome the complexities and costs of implementing a comprehensive DHT system in Vietnam. This should involve collaboration between the ministry of health and other relevant government departments (ie, the ministry of science and technology and the ministry of information and communications), as well as local and international collaboration with health care providers, the private sector, researchers, technology developers, social entrepreneurs, and consumers [67].

Fifth, efforts must be made to strengthen the governance of health-related cybersecurity***.*** The use of DHTs to record, store, and share health data increases the risk of privacy and security breaches [84]. Data security is a key concern for end users according to this review and is likely to affect the uptake of DHTs. Adequate governance of DHTs with regard to cybersecurity is therefore needed to protect users’ information and to promote trust in the security and integrity of DHTs. Currently, data protection laws in Vietnam are fragmented. Cyberinformation security (ie, information exchanged in a telecommunications or computer network environment) is governed by Law No. 86/2015/QH13 (2015), whereas cybersecurity falls under Law No. 24/2018/QH14 (2018) [85,86]. Although the law on cybersecurity recognizes *health* as an information system critical for national security, no detail on specific regulations or protections is provided with regard to health data in either of these laws. To promote trust, specific regulations concerning the protection of health data that are recorded, stored, and shared using DHTs are required. Resources must also be dedicated to enforcing the resultant regulations.

Sixth and last, we found that a lack of policies and protocols to guide DHT implementation and use was a common barrier to the application of DHTs to support patient-centered care. Conversely, clear positions and policies at the government level promoted confidence in DHTs within the broader community and guided their appropriate use. The Vietnamese government therefore has the opportunity to lead the way in DHT uptake in the nation. Developing and publishing guidelines on the use of DHTs, including patient-centered DHT-supported care, would set the standard for quality use of DHTs and inform best practice for both health care professionals and technology developers. Vietnam could look to neighboring countries as an example. In India, the ministry of health and family welfare collaborated with the Medical Council of India to develop telemedicine guidelines [38]. These guidelines supported the health care system to adapt quickly to telehealth during the COVID-19 pandemic while still maintaining a consistent standard of patient-centered care. Without such guidelines, many disparate approaches to telehealth could have resulted, and the quality of DHT-supported care would have been likely to vary.

### Limitations

This study may have been limited in its ability to provide a comprehensive overview of the use of DHTs to support patient-centered outcomes in all LMICs in the APR. Although the scoping review methodology allowed for a broad search, it was necessary to limit the search to papers published in English and Vietnamese. This may have excluded publications available in other regional languages.

### Conclusions

The use of DHTs is a viable option to increase equitable access to quality, patient-centered care across Vietnam and simultaneously reduce pressures faced by the health care system owing in part to a rapidly aging population and an increase in NCDs. Vietnam can take advantage of the lessons learned by other LMICs in the APR when developing its own approach to the use of DHTs. The following strategies are recommended: (1) emphasize stakeholder engagement, (2) strengthen digital literacy, (3) support the improvement of DHT infrastructure, (4) increase cross-sectorial collaboration, (5) strengthen governance of cybersecurity, and (6) lead the way in DHT uptake. Mapping existing DHT applications in Vietnam and evaluating the effectiveness of these DHT applications should be considered for future research. Investigations of the needs, preferences, and experiences of key Vietnamese stakeholders (eg, patients and their carers, health care workers and providers, and DHT developers) related to DHTs are also needed. This information would support the Vietnamese government to develop a national road map for DHTs and align the approach to DHT use most closely with local needs.

## Appendix

Multimedia Appendix 1 PRISMA-ScR (Preferred Reporting Items for Systematic Reviews and Meta-Analyses extension for Scoping Reviews) checklist.

Multimedia Appendix 2 Search strategy and results for MEDLINE.

Multimedia Appendix 3 Characteristics of the included studies.

## Abbreviations

APR: Asia-Pacific region
DHT: digital health technology
LMICs: low- and middle-income countries
mHealth: mobile health
NCD: noncommunicable disease
PRISMA-ScR: Preferred Reporting Items for Systematic Reviews and Meta-Analyses extension for Scoping Reviews
