# Supplementary material for: Lessons for Vietnam on the Use of Digital Technologies to Support Patient-Centered Care in Low- and Middle-Income Countries in the Asia-Pacific Region: Scoping Review
Source: J Med Internet Res. 2023 Apr 5;25:e43224. doi: 10.2196/43224 (PMC10132046; doi:10.2196/43224)
Supplement: Multimedia Appendix 2 [file jmir_v25i1e43224_app2.docx]

## Multimedia Appendix 2. Search Strategy and Results for MEDLINE

| **Search** | **Search Terms** | **Results** |
| --- | --- | --- |
| #1 | "bangladesh"  OR  "burma"  OR  "myanmar"  OR  "cambodia"  OR  "china"  OR  "fiji"  OR  "india"  OR  "indonesia"  OR  "laos"  OR  "malaysia"  OR  "maldives"  OR  "marshall islands"  OR  "micronesia"  OR  "mongolia"  OR  "nepal"  OR  "north korea"  OR  "pakistan"  OR  "papua new guinea"  OR  "peru"  OR  "philippines"  OR  "samoa"  OR  "solomon islands"  OR  "sri lanka"  OR  "thailand"  OR  "timor-leste"  OR  "tonga"  OR  "tukapu"  OR  "vanuatu"  OR  "vietnam"  OR  "Viet Nam" | 678,473 |
| #2 | “patient centred” | 7,524 |
| #3 | “patient centered” | 36,107 |
| #4 | “patient-centered care” * | 27,077 |
| #5 | “patient centric” | 1,338 |
| #6 | “patient focused” | 1,850 |
| #7 | “patient oriented” | 3,512 |
| #8 | “comprehensive care” | 4,613 |
| #9 | “comprehensive health care” * | 7,620 |
| #10 | “comprehensive healthcare” * | 328 |
| #11 | “holistic care” | 2,253 |
| #12 | “holistic medicine” * | 339 |
| #13 | “continuous care” | 1 |
| #14 | 2 or 3 or 4 or 5 or 6 or 7 or 8 or 9 or 10 or 11 or 12 or 13 | 53,577 |
| #15 | mhealth | 6,989 |
| #16 | m-health | 736 |
| #17 | telehealth | 9,431 |
| #18 | tele-health | 228 |
| #19 | “health informatics” | 4,186 |
| #20 | ehealth | 5,471 |
| #21 | e-health | 3,524 |
| #22 | “digital health” | 4,311 |
| #23 | digital-health | 4,311 |
| #24 | “Tele medicine” | 166 |
| #25 | Telemedicine | 40,693 |
| #26 | e-consultation | 98 |
| #27 | econsultation | 45 |
| #28 | eprescription | 28 |
| #29 | e-prescription | 106 |
| #30 | “e-health records” | 22 |
| #31 | “ehealth records” | 6 |
| #32 | “electronic health records” * | 35,229 |
| #33 | 15 or 16 or 17 or 18 or 19 or 20 or 21 or 22 or 23 or 24 or 25 or 26 or 27 or 28 or 29 or 30 or 32 | 58,747 |
| #34 | 1 and 14 and 33 | 28 |

* MeSH terms
