# Supplementary material for: Lessons for Vietnam on the Use of Digital Technologies to Support Patient-Centered Care in Low- and Middle-Income Countries in the Asia-Pacific Region: Scoping Review
Source: J Med Internet Res. 2023 Apr 5;25:e43224. doi: 10.2196/43224 (PMC10132046; doi:10.2196/43224)
Supplement: Multimedia Appendix 3 [file jmir_v25i1e43224_app3.docx]

## Multimedia Appendix 3. Characteristics of included studies

| **Reference** | **Aim** | **Study Design** | **Country, Setting, Participants, Health Condition** | **DHT(s)** | **NICE Classification** [30] | **Relevant Finding(s)** |
| --- | --- | --- | --- | --- | --- | --- |
| Adawiah et al., (2021) [69] | (1) What are the types of mHealth that have been used for CVD patient self-care? (2) What are the advantages of employing mHealth for CVD patient self-care? (3) What are the challenges in employing mHealth for CVD patient self-care? | Systematic review | Malaysia  N/A  N/A  Cardiovascular disease | mHealth | N/A | N = 2,095 articles identified, n = 19 included  **Outcomes:** (1) The usefulness of mHealth is identified through its various functions that improve patient’s knowledge, and confidence with their selfcare, instil positive attitude about their condition and using mHealth for selfcare, be active in symptom monitoring and recording, improve medication and appointment adherence, attain healthy lifestyle behaviour, record and share health data, and get motivated to exercise selfcare; (2) An integration of mHealth in patient’s self-care management has proven to reduce the rehospitalisation, emergency room visits and unplanned doctor’s visit (3) Majority of the studies have recorded a positive improvement in the clinical markers  **Enablers:** (1) Telerehabilitation enables personalised programmes tailored to the patient’s actual conditions, and enhances patient-professional interactions; (2) Integration of mHealth as part of telerehabilitation requires continuing support from healthcare professionals;  **Barriers**: (1) User anxiety due to technical issues, below expectation achievement, low technical literacy and continuous nudges; (2) User concerns about the accuracy of data or information; (3) Privacy concerns around information shared using mHealth, increasing perceived risk and reducing uptake; (4) Technical issues faced by users, especially older people; (5) Unequivocal outcomes caused by inconsistent engagement with mHealth |
| Alam et al., (2020) [72] | (1) To examine the factors affecting the adoption of mHealth services in Bangladesh by using the extended Unified Theory of Acceptance and Use of Technology (UTAUT) model with perceived reliability and price value factors; (2) To examine the moderating effect of gender on the intention to use and on the actual usage behavior of users of mHealth services | Quantitative - cross-sectional study with convenience sampling | Bangladesh  Hospital  n = 296 generation Y patients with previous experience of mHealth  Not described | mHealth | N/A | **Enablers:** (1) Performance expectancy, social influence, facilitating conditions and perceived reliability positively influence the behavioural intention to adopt mHealth services; (2) Effort expectancy and price value did not have a significance influence on the behavioural intention; (3) Gender has a significant moderating effect on mHealth services adoption in certain cases  **Policy/Practice:** Policy-makers should consider the roles of PE, SI, FC and PR in increasing the adoption of mHealth services. However, it is essential to focus particularly on the role of PE & FCs, which have the highest level of influence over mHealth usage intention |
| Ali et al., (2016) [33] | To assess the effectiveness of the ODK Scan paper-to-digital system during a testing period of three months | Mixed-methods - sequential explanatory design | Pakistan  Public–private mix model of the tuberculosis control program  N/A  Tuberculosis | Open Data Kit (ODK) Scan: an Android-based mobile application for document digitisation | Tier A: System services | **Outcomes:** (1) A significant time reduction in data aggregation and data transfer activities, but data verification and form-filling activities took more time; (2) field workers saw value in using ODK Scan, but they were more concerned about the time-consuming aspects of the use of ODK Scan  **Enablers:** Integrate application use within routine workflow, and providing minimal disturbance of existing practices, and continuous feedback  **Policy/Practice:** Devise a national-level policy, or at least organizational policy, for the integration of the mHealth technologies into public health programs to maximize opportunities to reach needy communities |
| Amudha et al., (2017) [74] | To describe the telehealth and telenursing status and its need in Indian context | Narrative review | India  N/A  N/A  Not described | Teleconsultation | N/A | **Outcomes:** (1) Telenursing is considered the best solution for extending the provision of essential healthcare throughout India, which is culturally and geographically diverse; (2) Telemedicine extends consultation and diagnostic facilities at low cost to remote areas through internet and telecommunications, and fills the rural-urban gap  **Enablers:** (1) Decreasing prices of electronic devices and its ease of use make the mobile technology as a suitable method in telemedicine projects in rural areas; (2) The launch of 4G and improved mobile technology infrastructure has helped the adoption of mobile health (mHealth)  **Barriers:** High cost of network and cost of incorporating telemedicine in network (difficult to adopt in low SES environments in India)  **Policy/Practice:** (1) The health care sector should leverage information technology to improve the quality of life, reduce human errors, reduce costs, and increase response timings and to achieve patient-centric health care systems; (2) There is a need for low cost delivery of healthcare at gross root level with continued telemedicine services |
| Balsari et al., (2018) [32] | To propose a federated, patient-centric, application programming interface (API)–enabled health information ecosystem that leverages India’s near-universal mobile phone penetration, universal availability of unique identification (ID) systems, and evolving privacy and data protection laws | Technical report | India  N/A  N/A  Not described | A federated, patient-centric, application programming interface (API)–enabled health information ecosystem | Tier A: System Service | **Policy/Practice:** (1) adopt a federated architecture; (2) prioritise patient and population health needs over billing needs; (3) guarantee a patient’s right to their structured data; (4) allow a plug and play model of highly customisable applications that can address varying context-specific needs, and that respond to market incentives for better user-interfaces; (5) mandate minimum data sets; (6) adopt privacy by design: automate audited and consented data flow; (7) defaul to safeguarding patients’ control over their data |
| Bateja et al., (2019) [57] | To propose a cloud-based recommender system/engine which will further integrate with user’s smartphone and wearable devices to prevent diabetes by recommending healthy lifestyle and diet to diabetic patients | Technical report | India  N/A  N/A  Diabetes | D-Care Recommender System: continuously monitors the user’s activity as the user logs the information, and applies ML based recommendations | Tier C: Inform clinical management | **Enablers:** (1) DHTs that monitor lifestyle factors and make preventative recommendations; (2) DHTs that integrate with the user's daily life and are user-friendly |
| Bilal et al., (2018) [61] | (1) To determine the relative inter-rater reliability and usability of standard and Mobile health (mHealth)-supported World Health Organization (WHO) algorithms for dehydration assessment in patients with acute diarrhea in a rural, low income country hospital (2) To investigate the reliability of the individual clinical signs of dehydration used in the WHO algorithm among children and adults with acute diarrhea | Quantitative - observational prospective diagnostic study with random sampling | Bangladesh  Hospital  n = 469 children and adults  Acute diarrhoea | Mobile health (mHealth)-supported World Health Organization (WHO) algorithms for dehydration assessment | Tier C: Diagnose a condition | **Outcomes:** mHealth technology improves the reliability of dehydration assessments conducted by nurses on patients presenting with acute diarrhea  **Policy/Practice:** mHealth may help further standardise clinical care for patients in resource-limited settings while potentially reducing both overutilisation and underutilisation of scarce health care resources, such as intravenous fluids and hospital admissions |
| Chew at al., (2020) [43] | To describe the usability and utility testing of a newly developed medication adherence app—MedAssist—among ambulatory care patients in Malaysia | Qualitative - in-depth interviews with purposive sampling | Malaysia  Primary care clinic  n = 13 ambulatory patients taking two or more long-term medications  Chronic conditions | MedAssist medication adherence app | Tier B: Health and care diaries | **Outcomes:** Participants found Med Assist easy to use and user friendly, as they were able to complete the tasks given to them. However, the details required when adding a new medication were found to be confusing despite displaying information in a hierarchical order. Participants who were caregivers as well as patients found the multiple-user support and pill buddy utility useful. This suggests that Med Assist may improve the medication adherence of patients on multiple long-term medications  **Enablers:** The usability and utility testing of Med Assist with end users made the app more patient-centred in ambulatory care |
| Damani et al., (2020) [38] | (1) To describe the challenges, approaches to solutions with evidence-based practices that can be utilised to ensure competent management of cancer pain during the COVID-19 pandemic in India  (2) To provide an overview of adapting to telehealth consultations for identification, evaluation and management of cancer pain, safe and rational use of analgesics and adjuvant drugs, recognising and responding to holistic care needs and addressing the total pain, ensuring continuity of pain management, and strategies when complying with narcotic drug regulations, while ensuring safety of patients and HC providers | Qualitative study – not further described | India  Tertiary cancer hospitals  Pain and palliative care teams  Cancer pain | Teleconsultations | Tier B: Communicating about health and care | **Outcomes:** With appropriate adaptations, teleconsultation can be an effective way of providing quality cancer pain management  **Enablers:** (1) Triaging of patients who are best suited for telehealth; (2) Choice of appropriate telehealth platform (video-based telehealth enables empathy and psycho-social-spiritual support; better for getting to know the patient)  **Barriers:** (1) Unfamiliarity, technological and technical difficulties; (2) Very low income or rural patients may not have access to mobile phones and/or internet connection; (3) The development of rapport is more difficult than when consulting in-person; (4) Extra efforts are needed to develop adequate systems for scheduling telephonic appointments and for satisfactory documentations  **Policy/Practice:** (1) Indian Ministry of Health and Family Welfare in collaboration with Medical Council of India (MCI) developed telemedicine guidelines to enable timely, quick, effort-saving and cost-saving healthcare provision during an emergency (e.g. COVID-19) [these were adapted for treatment of cancer pain in this context]; (2) Clinical practice guidelines were used to triage patients unsuitable for telehealth |
| Dang et al., (2021) [67] | To explore the opportunities, challenges, and necessary conditions for Vietnam in transforming toward a patient-centred care model to produce better health for people and reduce health care costs | Narrative review | Vietnam  N/A  N/A  Not described | Bespoke Healthcare System (BHS) | N/A | **Outcomes:** (1) Clinical access to patients’ real-time health information would enable tailored precision and holistic health solutions for individuals; (2) Electronic prompts and reminder alerts have been shown to assist individuals in adhering to clinical intervention effectively, particularly in managing long-term chronic conditions; (3) Patients would optimally be equipped with a comprehensive understanding of their conditions, treatment options, and self-management strategies; (4) Clinicians would be provided with up-to-date, evidence-based optimal health care pathways suitable for their specific patients’ conditions; (5) The platform might be used to upskill patient families and carers, which would strengthen community care and lessen the health care burden due to staff shortages  **Barriers:** (1) The high cost and complexity of implementing digital health information systems, such as EMRs, may be a barrier to broad dissemination; (2) The digitalization of data and services represents a potential cybersecurity threat to privacy and trust of people in a new health care system  **Policy/Practice:** (1) Successful adoption of the BHS requires a whole-system approach involving the support of different sectors in the society; (2) The Vietnamese government needs to improve the security of information technology platforms in general and health care in particular to protect patient privacy and information security; (3) To improve the adoption of evidence-based practices, it will be necessary to provide resources to demonstrate the effectiveness and impact of digital health initiatives and establish a network of collaborators including health care administrators, clinicians, community representatives, digital health researchers, information technology developers, and public health education experts; (4) To increase the acceptability and feasibility of digital health initiatives, a co-design approach is crucial |
| Fan et al., (2021) [60] | To understand how health chatbots are used in a real-world context, what issues and barriers exist in their usage, and how the user experience of this novel technology can be improved | Mixed-methods - descriptive analysis and content analysis | China  Community  n = 16,519 chatbot users  Not described | Self-diagnosis chatbot (DoctorBot): an AI-driven, mobile based medical consultation platform | Tier C: Diagnose a condition | **Outcomes**: (1) Users spanned all age groups, including middle-aged and older adults. Users consulted the chatbot on a wide range of medical conditions, including those that often entail considerable privacy and social stigma issues; (2) There were two prominent issues in the use of the chatbot: (1) a considerable number of users dropped out in the middle of their consultation sessions, and (2) some users pretended to have health concerns and used the chatbot for nontherapeutic purposes  **Barriers:** Insufficient actionable information and  perceived inaccurate diagnostic suggestions  **Policy/Practice:** Designers and developers should employ user-centred approaches to address the issues and user concerns to achieve the best uptake and utilisation, i.e. ensure ease of use, increase accuracy and clarity of recommendations, provide onboarding material to introduce users to the chatbot and let them know what to expect |
| Faujdar et al., (2021) [31] | To assess the perceptions of various stakeholders about the implementation of IHIS4PHC in order to propose a model of behaviour change for eHealth using the theory of interpersonal behaviour (TIB) and the capability, opportunity, and motivation from behaviour (COM-B) change wheel | Qualitative - in-depth interviews and focus group discussions, and participatory observation | India  Primary care centre  n = 20 patients; n = 6 healthcare workers  Hypertension, diabetes, antenatal/postnatal care, acute conditions (not described) | Integrated Health Information System for Primary Health Care (IHIS4PHC) | Tier A: System service | **Outcomes:** (1) Healthcare workers highlighted that working with the digital health system was initially arduous, but they later realised its usefulness, as the digital system made it easier to search records and generate reports, rapidly providing evidence to make decisions; (2) Auxiliary nurse midwives reported that recording information on computers saved time when generating reports; however, systematic and mandatory data entry made recording tedious; (3) Staff were apprehensive about the use of computer-based data for monitoring their work performance; (4) Patients appreciated that their previous records were now available on the computer for easy retrieval  **Enablers:** On-the-spot technical support to ensure uninterrupted work  **Barriers**: Handling of IT equipment, interrupted electrical supply, and fears of a computer system breakdown |
| Ghozali et al., (2021) [59] | To describe the systematic design, development, and implementation process of a Google Android OS asthma self-management smartphone app according to the Patient Centered Design approach | Technical report and pilot study | Indonesia  Primary care  n = 27 patients  Asthma | AsmaDroid mHealth app | Tier C: Inform clinical management | **Outcomes:** A usability study of the app showed that the average success rate was as follow: “completed with ease” was 88.15%, while “completed with difficulty” was 7.78%, and “failed to complete” was 4.07%. It means that the app has a “very high” success rate as higher than 78%. Other categories “completed with difficulty” and “failed to complete” were lower than 10%, meaning that it was normal for users to make errors when performing tasks  **Enablers:** Involvement of end-users in the design to DHTs to ensure they meet users' expectations and needs |
| Guo et al., (2020) [49] | To determine whether a mobile health (mHealth) technology-supported AF integrated management strategy would reduce AF-related adverse events, compared with usual care. | Quantitative - cluster randomised controlled trial | China  mAFA-II trial centres  IG: n = 1,646 patients; CG: n = 1,678 patients  Atrial fibrillation (AF) | mAFA platform to manage patients with AF | Tier C: Inform clinical management | **Outcomes:** (1) Rates of the composite outcome of ‘ischemic stroke/systemic thromboembolism, death, and rehospitalization’ were lower with the mAFA intervention compared with usual care (1.9% vs. 6.0%; hazard ratio [HR]: 0.39; 95% confidence interval [CI]: 0.22 to 0.67; *P*<.001); (2) Rates of rehospitalization were lower with the mAFA intervention (1.2% vs. 4.5%; HR: 0.32; 95% CI: 0.17 to 0.60; *P*<.001); (3) Subgroup analyses by sex, age, AF type, risk score, and comorbidities demonstrated consistently lower HRs for the composite outcome for patients receiving the mAFA intervention compared with usual care (all *P*<.05) |
| Gupta et al., (2021) [35] | To compare the outcomes of health services utilised by the patients in terms of pre‑hospital care and referral to tertiary care hospital before and after the implementation of telemedicine program | Mixed-methods - cross-sectional with an interpretive case study approach | Nepal  Rural telemedicine program implementation sites  n = 315 patients  Respiratory, genitourinary, musculoskeletal, skin, ENT, gastroenterology, dental, eye, cardiovascular and CNS conditions | Telemedicine | Tier B: Communicating about health and care | **Outcomes:** (1) Significant decrease in the number of referrals from the centre where telemedicine is implemented. The reason is most likely the better identification of patients who actually require a referral, thanks to the expertise of the specialist working in tertiary care centre; (2) The average time taken to reach the telemedicine centre were 25 min, whereas it took 75 min to reach the nearest district hospital  **Enablers**: Presence of specialists during telehealth to provide care and recommendations in more complex cases (reduces the need for referrals)  **Policy/Practice:** Telemedicine that connects rural health centres to specialist medical professionals can reduce referrals to tertiary care hospitals, which improves health outcomes in a cost-effective way |
| Gupta et al., (2021) [62] | To investigate the benefits of tele-otology in community screening of patients with ear diseases | Quantitative - retrospective study | India  Hospitals and clinics  n = 810,746 patients screened and treated under the Shruti tele-otology program  Ear diseases (e.g. impacted wax, chronic suppurative otitis media, diminished hearing, acute otitis media, acute suppurative otitis media, foreign body, otomycosis) | Telemedicine device (ENTraview), a camera-enabled android phone integrated with an otoscope and audiometry screening | Tier C: Diagnose a problem | **Outcomes:** (1) Ear problems were found in 265 615 (33%) patients, of which 151 067 (57%) had impacted wax, 46 792 (18%) had chronic suppurative otitis media, 27 875 (10%) had diminished hearing, 12 729 (5%) had acute otitis media and acute suppurative otitis media (ASOM), and 27 152 (10%) had problems of foreign body, otomycosis, and so on. Of the total 265 615 referred patients, 20 986 (8%) reported for treatment and received treatment at a significantly reduced cost through Shruti program partners; (2) Forty-five percent patients reported a significant improvement in their quality of life; (3) Provides ear care at a significantly lower cost |
| Hossain et al., (2019) [71] | To evaluate the current evidence on digital interventions for people living with NCDs (non-communicable diseases) in India and the outcomes of those interventions | Systematic review | India  N/A  N/A  Non-communicable diseases (diabetes, mental health disorders, cerebral palsy, metabolic syndrome) | Interventions delivered through mobile phone, computer, internet, tablet, social media, and any digital medium | N/A | **Outcomes:** Investing in digital health can reduce the cost of direct care, enhance access to health services, and improve health outcomes for resource-constrained countries  **Barriers:** DHTs that only focus on one health condition and are not tailored to patients with multiple comorbidities  **Policy/Practice:** (1) Rural areas are likely to have a higher need for DHTs due to lack of health infrastructure, few specialists, and lack of access to health services. Interventions should focus on rural areas to reduce urban-rural health disparities; (2) Consider measures to increase sustainability: subsidised access to digital health devices and services; incentivising healthcare providers to use digital health; development of low cost, culturally appropriate digital interventions for different NCDs; (3) There is a need for protection of personal health data through legislative and regulatory measures, and appropriate protection of personal data from data theft; (4) Collaboration between multiple stakeholders to promote digital healthcare |
| Huang (2015) [45] | RQ1: What do these hospitals have in common in their Web development?; RQ2: What problems and challenges are these hospitals facing in their Web development?; RQ3: How have these hospitals excelled in serving their patients online? | Qualitative - in-depth interviews and content analysis. Purposive sampling | China  Hospital  Not described  Not described | Hospital websites | Tier B: Promoting good health | **Enablers:** Website developers should consider inclusion of patient-centred features: (1) At least 70% of the links that directly pertain to patients in above-the-fold space on the home page (2) At least 10 interactive tools (3) At least 20 e-health videos for patients (4) A personalised patient portal (5) A menu that contained interactive tools (6) A menu or a section that contained patient-related videos |
| Jiang (2019) [41] | To investigate whether good quality of communicative experiences at the point of care may motivate patients to communicate with providers via the Internet at a later time point, and how this relationship may be moderated by patient trust and patient satisfaction, in the context of China | Quantitative - descriptive survey | China  Not described  n = 644 adults aged 40+ who visited a doctor or doctor’s office during the past 12 months  Not described | Online patient-provider communication | Tier B: Communicating about health and care | **Outcomes:** (1) Face-to-face patient-provider communication had a positive and direct effect on online patient-provider communication at a later time point (2) Patient trust and patient satisfaction both positively moderated the relationship between face-to-face and online patient-provider communication  **Policy/Practice:** (1) To motivate patients to use new technologies for consultation, health care organizations must attend to the development of trusting relationships with their patients and the enhancement of quality care to foster deep loyalty that leads to proactive patients who would continue to receive health care services via the Internet (2) Patient education with a focus on improving knowledge and skills of online medical communication is critically needed |
| Keikhosrokiani et al., (2018) [54] | To investigate healthcare professional’s opinions [about iHeart] and to find success factors of such a system before its full implementation | Quantitative - descriptive survey | Malaysia  Healthcare setting  n = 169 healthcare professionals  Coronary heart disease | iHeart – a wearable monitoring device that measures blood pressure and heart rate | Tier C: Inform clinical management | **Enablers:** usability, communicability, data processing, time, urgency, currency, accuracy, reliability, system support, assurance, trustworthiness; Users must have literacy of using iHeart in order to be satisfied and the system becomes successful. Therefore, instruction must be provided for the users before they use the patient-centric healthcare system  **Policy/Practice:** The system must have a separate design based on the culture and strategic position as the people point of view and facilities are different among different nationalities |
| Krishnan et al., (2015) [52] | To develop a theoretical model representing the factors that influence the adoption of Consumer Health Informatics (CHI) applications amongst consumers in Malaysia | Quantitative - cross-sectional study with convenience sampling | Malaysia  Community, hospitals and clinics  N = 105 health consumers  Major or minor illness (not described) | Consumer Health Informatics (CHI) applications | Tier C: Inform clinical management | **Enablers:** Use of DHTs that are fun or pleasurable to use (increases motivation); Use of DHTs that consumers find easy to use and understand; Use of DHTs that consumer perceives will help them to attain their health self-management goals, unobtrusive and fit with the user's lifestyle, minimise the need for manual data entry  **Policy/Practice:** Developers and policy makers should consider the behavioural factors that influence consumers' intentions to adopt DHTs, as these can influence the success of DHTs |
| Kumar et al., (2020) [72] | To explore the psychological mechanism that determines the e-Health services continuance usage intention through application of technology acceptance model (TAM) and expectation-confirmation model (ECM) | Quantitative - cross-sectional study with purposive sampling | India  Hospital  n = 453 outpatients and caregivers who had used e-Health services at least once  Not described | e-Health services (appointment scheduling, health information search, online payment, telemedicine, electronic health records, and others) | N/A | **Outcomes:** (1) The direct relationship among perceived service quality, satisfaction and usage intention is found to be significant; (2) Social influence has a positive effect on the e-Health service usage  **Enablers:** Perceived privacy and security influence trust and user satisfaction - only when hospitals achieve a high level of patient confidence in security and privacy do users trust hospitals and use e-Health services continuously. Trust will result in directly and indirectly affecting usage intention through satisfaction |
| Lai et al., (2021) [37] | To investigate the policy interventions, development trends, and service innovations of internet hospitals in China | Qualitative - document analysis and interviews with purposive sampling | China  Not described  n = 58 key stakeholders (policy  makers, not-for-profit professional societies, hospital administrators, doctors, managers of pharmaceutical companies, managers of internet companies, information technology engineers, academic researchers, and financial investors)  Not described | Internet hospitals (encompassing telemedicine, electronic prescriptions, medical insurance, commercial health insurance, health management, hospital operation, and hospital logistics) | Tier B: Communicating about health and care | **Outcomes:** (1) Internet hospitals might offer three distinctive values: saving patients' and their families' time, providing more convenient access to high quality medical resources and expanding from treatment oriented services to complete health management; (2) Internet hospitals can reinforce the interactions between physicians and patients without the challenges of geographical limitations; increase the accessibility of high-quality medical resources in remote and rural areas; and dramatically reduce the indirect costs of medical care for patients, especially the expenses and time associated with travel; (3) Internet hospitals can provide patients with more convenient methods for accessing high-quality medical resources and save patients’ and their families’ time  **Policy/Practice:** There is a need to introduce specific medical insurance measures at the national level as implementation guidance for local governments |
| Lim et al., (2021) [50] | To evaluate the feasibility of a COVID-19 symptom monitoring system (CoSMoS) by exploring its utility and usability with end-users | Qualitative - in-depth interviews with purposive sampling | Malaysia  Hospital and community  n = 11 patients with suspected COVID-19 infection and n = 4 doctors  COVID-19 | COVID-19 symptom monitoring system (CoSMoS): remote home monitoring for patients with suspected COVID-19 infection | Tier C: Inform clinical management | **Outcomes:** CoSMoS was useful in providing close monitoring and continuity of care, supporting patients’ decision making, ensuring adherence to reporting, and reducing healthcare workers’ burden during the pandemic  **Enablers:** (1) DHTs that provide a communication channel between patients and HCPs, allowing patients to express their concerns; (2) Triaging of patients based on risk/severity, which is both automated and closely supervised by doctors; (3) Providing patients with manual and technical support (improves the adoption of DHTs; (4) The development of a telemonitoring application for an emerging communicable disease should be agile and adaptable to the rapidly changing clinical evidence  **Policy/Practice:** Usability exploration is important to get end-users views and experience to improve the system further (i.e. co-design) |
| Mahajan et al., (2020) [65] | To discuss issues related to using telemedicine during the SARS-CoV-2 pandemic | Narrative review | India  Not described  Paediatrics  COVID-19 | Telemedicine | N/A | **Outcomes:** Time saving; Easy accessibility; Social distancing; Triage; First aid; Refill prescriptions; Cost effective; Reduces overcrowding; Health education; Using telemedicine, it is possible to provide a more holistic care faster e.g., we can take advice from the expert in a shorter duration without referring the patient for expert opinion. Services such as tele-radiology, tele-pathology will also aid us in faster diagnosis. Common procedures (such as use of metered dose inhalers, technique of giving insulin injections) can be shared with the patient/caregiver via YouTube links, pre-prepared videos or live demonstrations  **Enablers:** (1) Good quality internet connections; (2) Uninterrupted power supply; (3) Workshops on telecommunication; (4) Providing consultations via video and providing prescriptions via email can assist with correct interpretation of information  **Barriers:** (1) DHTs can make physical examination difficult or impossible (may lead to underestimation or misinterpretation of illness); (2) Lack of widespread access to telecommunication facilities to the wider public leads to inequitable access to health services via telemedicine; (3) Level of patient education, SES or health literacy can limit patients' ability to engage with telehealth and/or to act on advice or prescriptions provided via telehealth  **Policy/Practice:** (1) Designated centres such as post offices, dispensaries and primary health care centres where good internet services and trained facilitators are available. The staff should be trained in performing video calls and explaining prescription to the patients; (2) Procedures to protect patient confidentiality must be in place |
| Maniam et al., (2015) [51] | (1) To identify factors that influence the intention to adopt DSMA by diabetics in Malaysia (2) To develop a research model to represent the adoption of DSMA amongst diabetics in Malaysia | Quantitative - cross-sectional study with convenience sampling | Malaysia  Community  n = 105 people  Diabetes | Diabetes Self Management Applications (DSMA) in the form of smartphone, web-based and desktop-based applications | Tier C: Inform clinical management | **Barriers:** Perceived Financial Risk (fear of wasting money towards to DSMA); Perceived Privacy and Security Risk (fear of sharing private data through DSMA); Technology Anxiety (tendency of feeling uneasy, apprehensive, or aversive at the prospect of using DSMA); Facilitating Conditions (degree to which a patient believes they have the necessary resources, knowledge and skills to use of DSMA) |
| Naveen et al., (2022) [39] | To prospectively validate 2 novel task–based PCOMs for evaluation of upper and lower extremity physical function, namely arm lift (AL) test and two-minute walk distance (2MWD), respectively, in adults with idiopathic myositis (IIM) | Quantitative - cross-sectional observational study | India  Rheumatology tertiary-care centre  n = 22 adults  Idiopathic myositis (IIM) | Telemedicine, tele‐rheumatology | Tier B: Communicating about health and care | **Outcomes:** (1) Patient-centred assessments like AL allow more regular and frequent measurements by patients at home; (2) Novel PCOMs, particularly arm-based assessments, may be used for remote assessment of muscle strength, as a refection of disease activity, pending validation in larger cohorts of IIM  **Enablers:** DHTs that have been validated for use in remote health settings |
| Nazir and Soroya (2021) [44] | To examine the use of the internet and social networking sites for health-related information seeking | Quantitative - cross-sectional study with purposive sampling | Pakistan  Diabetes and cardiac centre  n = 200 patients  Diabetes or hypertension | Internet-based information sources including search engines, medical websites, blogs, email, e-newspapers and social media websites | Tier B: Promoting good health | **Outcomes:** (1) WhatsApp, YouTube and Facebook were the most commonly used sources of health-related information; (2) Smartphones were the most frequently reported device used to seek health-related information on the internet; (3) Patients sought health-related information on the internet once per week on average  **Barriers:** (1) Unreliable internet services (connectivity, network coverage, speed); (2) Difficulty determining the credibility/reliability of available information; (3) Difficulty understanding the information available; (4) Information the person sought was not available on the internet |
| Nedungadi et al., (2018) [58] | To describe the architecture and preliminary validation of a system that management the health of patients in remote and rural villages | Technical report | India  Community (remote and rural villages)  N/A  Not described | AmritaJeevanam (platform consisting of multiple invasive and non-invasive sensors, an interface unit and a personalised health monitoring and awareness application) | Tier C: Inform clinical management | **Outcomes:** (1) Pilot testing showed system accurately determines patient conditions based on vitals with the battery lasting for 16 h in the active mode. Survey feedback indicates that the 82% of patients were able to easily operate and understand the guidance provided by the system. Over 94% of the patients found value in the personalised feedback; (2) Enables semiliterate and rural patients who lack easy access to health care to utilise the easy-to-use system to monitor basic conditions, increase understanding and awareness of health conditions, and reduce emergency conditions  **Enablers:** (1) Low overall cost; (2) Available in local languages; (3) Consideration of device/system performance with regards to computational power and battery life; (4) Use of differential diagnosis algorithms to provide patient-centric personalised health monitoring; (5) Inclusion of personalised preventative information relevant to the patient's specific health conditions (e.g. diet, exercise) |
| Nie (2017) [46] | To explore (1) individual preferences for the types and amounts of health information and decision-making autonomy among Chinese patients with T2DM; (2) their use of mobile technology in their self-management of the condition; and (3) the relationship between their use of mHealth and their preferences for health information and decision-making autonomy | Quantitative - descriptive study | China Hospital  n=200  People with diabetes | mHealth (health-related information online and health-related apps) | Tier B: Promoting good health | **Policy/Practice:** (1) When addressing issues relating to specific health conditions, treatment, laboratory tests, CAM, psychosocial aspects, and healthcare providers, healthcare professionals should be sensitive to the patient’s desire for a greater role in decision-making and tailor their communications accordingly; (2) Policymakers need to consider gender and education levels when create the polies both to encourage male patients to seek information and participant in decision-making, and to meet the needs patients with low education levels. Thus, mobile technology policymakers need to consider the feasibility and usability of mobile technologies; (3) Policymakers should develop standards for the health information available via mobile technologies and then create policies that encourage the industry to monitor that health information and ensure that it meets those quality standards; (4) Healthcare and mHealth policymakers need to consider the population who do not have Internet access, do not have a smartphone, or have a smartphone but do not know how to use smartphones to access health information |
| Pai and Alathur (2019) [66] | To propose a mobile health system framework for managing health and wellness among Indian populations | Qualitative - interviews with convenience sampling, and literature review | India  Not described  Residents (students, academicians, and other citizens), healthcare professionals, technology entrepreneurs, and software developers working in the field of health information management and mobile health systems  Not described | mHealth | N/A | **Outcomes:** (1) Allows patients immediate access to healthcare - in both rural and urban settings; (2) Assists with appointment reminders; (3) Reduces repeated patient interaction/assessment; (4) Allows timely access to patient records at time of treatment; (5) Healthcare can be delivered with minimum human resources; (6) Assists patients to identify and schedule available appointments; (7) Enables health professionals to remotely monitor signs and symptoms  **Enablers**: (1) Building strong relationships between patients and providers; (2) Public education and ease-of-use of technology facilitates uptake by patients; (3) Use of applications that are tailored to a patient's individual needs; (4) Use of a secure cloud-based patient information database that can be accessed by patients and HCPs  **Barriers:** (1) Patients may feel increased anxiety when using phone-based services compared to face-to-face; (2) Low literacy can limit patients' understanding of written information on a digital platform; (3) Patients may under-report or give biased responses when self-reporting, potentially reducing effectiveness of care given through mobile health  **Policy/Practice:** (1) Governments and healthcare services need to positively promote mobile health to increase acceptance amongst patients; (2) Policies must address potential trust and privacy issues (e.g. prevention of data theft); (3) Adequate health infrastructure and coordination within and between health services is needed for adoption of mobile health |
| Shrivastava et al., (2020) [73] | To discuss security and privacy challenges identified for seamless development of the components of the planned Federated Digital Healthcare Ecosystem in the Indian context | Narrative review | India  N/A  N/A  Not described | e-Governance applications | N/A | **Policy/Practice:** Policies must consider security and privacy challenges including (1) securing hybrid systems i.e. EHR facilities with video capabilities of third party platforms, (2) balancing protection of public health and personal privacy, (3) consent may not be a viable consideration for all the purposes, (4) anonymised tracking and contact-tracing mechanisms, (5) collection of health data by non-healthcare providers, (6) transparent data sharing, (7) protection against both cybercrimes and unauthorised sharing and (8) seamless integration of makeshift services as well as scaling up of healthcare service delivery by the ecosystem |
| Sini et al., (2015) [63] | To assess the use of mobile obstetric monitoring (MOM) to facilitate a public-private partnership of midwives and ObGyns | Quantitative - prospective cohort study with random sampling | Indonesia  Community  n = 656 women  Pregnancy | Philips Mobile Obstetrics Monitoring (MOM) software | Tier C: Diagnose a condition | **Outcomes:** Allowed the team to – (1) Focus on the requirements of the pregnant women and tailor management based on their needs (2) Review pregnancy records and take appropriate decisions in a timely manner (3) Facilitate appropriate referrals; Study participants almost unanimously reported a feeling of safety of being remotely monitored by the ObGyns and more time for discussion with the ObGyns  **Enablers:** DHTs that facilitate a team-based approach between healthcare professionals |
| Sudhahar et al., (2010) [42] | To investigate the applicability of an eSolution that improves healthcare delivery settings in the rural parts of the developing world | Quantitative - pilot study | Sri Lanka  Hospital  Hospital patients  Not described | Electronic Medical Records (EMRs) and video-based teleconsultations | Tier B: Communicating about health and care | **Outcomes:** Significant reduction in total travel cost (90.94%), in travel distance (80%) and travel time (75%)  **Enablers:** DHTs with adequate protection of patient health record with limitations at all levels |
| Sun et al., (2021) [64] | To introduce the diabetic M-healthcare system and describe progress in implementation for remote and mobile healthcare in China | Narrative review | China  N/A  N/A  Diabetes | mHealth | N/A | **Outcomes:** (1) The M-healthcare system compensates for incomplete medical information gathered during traditional treatment, making up for medical data gaps; (2) Patients diagnosed with diabetes often express the need to acquire diabetes-related medical knowledge. The diabetes health education network multimedia teaching platform can provide such knowledge. This teaching network platform can provide audiovisual education for patients with diabetes at home or on the hospital ward. The diabetes health education network platform also includes a special communication platform, allowing interactions among patients and between patients and the medical team to improve self-management and compliance; (3) M-healthcare is a promising method to bridge the gap between supply and demand for rural medical services |
| Sun et al., (2021) [70] | To describe clinical applications of mHealth for foraging-related neurological diseases, and the problems and challenges that mHealth faces in the management of these diseases | Narrative review | China  N/A N/A  Foraging-related neurological diseases | mHealth | N/A | **Enablers:** (1) Use of DHTs that reach patients in their home evironment (e.g. telerehabilitation) - makes interventions more tailored (thus potentially more effective), increase patient participation and patient satisfaction; (2) Adaption of DHTs (e.g. smartphone apps) to different culutral backgrounds and education levels; (3) Effective triaging systems that identify patient's needs and direct to the right form of care (increases continuity of care and care efficiency)  **Barriers:** Use of DHTs that only collect simple data without additional support or interpretation from healthcare professionals  **Policy/practice:** (1) There is a need for a standardised approach or policy regarding mHealth at the government level; (2) Data sharing between hospitals/healthcare systems is needed to reduce repeated patient interactions and increase efficiency; (3) A standard operating model within a single health service is important for maximising efficiency; (4) Creation of an international medical alliance that connects HCPs from LMICs to those from developed countries in order to share resources |
| Tan et al., (2020) [40] | To explore the attitudes and preferences of mental health service stakeholders regarding mobile mental health services and discuss the challenges and opportunities faced by mobile technology developers in China | Quantitative - descriptive cross-sectional study with convenience sampling | China  Not described  n = 586 (184 patients or their family members, 225 mental health professionals, 177 people from the general population)  Mental health conditions | Mobile mental health services | Tier B: Communicating about health and care | **Enablers:** (1) Professional support has proved to be the most critical facilitator of user acceptability because both face-to-face and professionally guided web-based services are preferred over unguided self-help services; (2) To reduce the threshold and obstacles for the public to positively seek and accept mental health services, professional mobile platforms could be equipped to establish more coherent service flows; (3) Mobile mental health services should be integrated into people’s daily life habits as much as possible through common social media such as WeChat or QQ, which are preferred platforms for service demanders  **Barriers:** (1) Low e-mental health awareness (e-awareness) and the digital divide of patients with severe mental illness might hinder the future use and perceived helpfulness of mobile services; (2) Time consumption and workload disruption might hinder the sustainable willingness of more professionals to provide such services  **Policy/Practice:** (1) Service developers should pay more attention to the different needs of different stakeholders and try to meet these differentiated needs in a more acceptable manner; (2) Service providers need to come up with more individualised and authorized health education, which can not only guarantee the effectiveness of information but also help information seekers avoid being inundated and misled by the massive amount of information |
| Tuli et al., (2016) [56] | To present our work in progress – Harmony - a system designed to encourage the patients in adopting an efficient morning routine | Technical report | India  Psychiatric department of AIIMS  n=4 caregivers, n=3 psychiatrists and n=2 members of clinical staff from AIIMS, n=2 computer science researchers from IIITD  Serious mental illnesses (SMIs) | Harmony - a system composed of (a) an Android application for patient; (b) an Android application for caregiver; and (c) a web dashboard for clinical usage by doctors | Tier C: Inform clinical management | **Enablers:** (1) Features to promote medication adherence; (2) Features to track self-improvement (3) Connectivity with doctors; (4) Application specifically for caregivers; (5) Able to be accessed remotely/offline; (6) Real-time appointment scheduling  **Policy/Practice:** Developers should consider involving end-users in the development of DHTs to ensure they meet their needs and expectations |
| Verma et al., (2021) [68] | To highlight the voluminous advancement of technology rendering support to achieve a digitized health care future. With the help of the research done and a constant upgradation of technology, the authors of this study would like to bring your attention to the journey of telemedicine and the future of healthcare system with technology advancement | Systematic review | India  N/A  N/A  Not described | Telemedicine | N/A | **Outcomes:** Telehealth increases the provision of healthcare services in remote and low SES area areas, thus increasing the patient access to health care facilities and reducing costs to patients  **Enablers:** (1) A proper technical infrastructure, data storage systems and technical support are required for a proper functioning of telemedicine; (2) Cultural factors - willingness to adapt and a positive attitude towards change amongst all the stake holders is, therefore, one of the main pre-requisites needed for a smooth functioning and development of technology; (3) User’s perception with respect to interactivity, data security, usefulness, trust, ensuring privacy influences the adjustment of telemedicine for regular utilisation in health care practice  **Barriers:** Lack of appropriate training and protocols for HCPs makes it difficult to perform triage, establish a correct diagnosis and to communicate with the patients  **Policy/Practice:** To overcome the primary challenge of integrating telemedicine in the main stream health care delivery system, standardized guidelines for health care procedure as well as infrastructure need to be formulated |
| Vitale et al., (2016) [53] | To investigate the differences in intermediate health outcomes between two diabetes care programs: one a comprehensive diabetes centre, the other a limited care setting | Quantitative – cross-sectional study | India Jothydev’s Diabetes and Research Centre, Kesavadev Trust Basic Diabetes Care Centre and Kesavadev Trust diabetes treatment camps (Diabscreen Kerala Camps) CG: n=75; IG: n=100 Diabetes | Diabetes Tele-Management System (DTMS) - comprises a system for patients to report BG values which are analysed by the multidisciplinary team (with dosage adjustments and lifestyle changes able to be recommended within 24hr); diabetes education for patients; continuous monitoring of multi-drug compliance, glucose monitoring, insulin injection technique, and lifestyle; reminder calls | Tier C: Inform clinical management | **Outcomes:** (1) HbA1c (7.62 vs. 8.58, p = 0.003), cholesterol (134.4 vs. 173.4, p < 0.001), and diastolic blood pressure (72.9 vs. 77.0, p = 0.016) were significantly lower in patients receiving comprehensive care, while the reductions in systolic blood pressure (134.6 vs. 138.7, p = 0.202) did not achieve statistical significance; (2) The comprehensive care group achieved better intermediate health outcomes than the limited care group; (3) Patients in limited care checked their BG levels less frequently, were less likely to own a glucometer, and were less likely to use insulin than patients in the comprehensive care setting; (4) Subjects receiving comprehensive care had better intermediate health outcomes not because they were wealthier than the limited care subjects at baseline, but because they are receiving a different level of diabetes care; (5) These outcomes, which remained significant after correcting for confounding factors, could be attributed to more aggressive treatment regimens in the comprehensive care centre, as well as the real-time, frequent communication with medical professionals in the telemedicine program |
| Vorakulpipat et al., (2019) [34] | To present a tool, called the EasyHos system, as a strategic resource to reduce the unexpected problem at hospitals | Technical report | Thailand  Hospital  Not described  Not described | EasyHos mobile phone app for improving patient flow in hospitals | Tier A: System service | **Outcomes:** EasyHos lead to reductions in (1) The number of times patients requested information from hospital staff (e.g. about their appointment status) (2) Amount of time staff spent seeking information and responding to patients, allowing them more time to complete their routine tasks (3) Patients not being present in the waiting room when their turn is called, increasing efficiency  EasyHos allows patients to access the information they need immediately; EasyHos has low or nearly zero implementation costs |
| Wang et al., (2020) [47] | To design a series of patient-centred health education messages for patients with T2DM, based on American Diabetes Association on Taking Care of Diabetes, following a standard process and then evaluate its effect of the management model by SMS to provide an economic and effective model for elderly diabetes management in poor areas | Quantitative - two-arm randomized controlled trial | China  Hospital  CG: n=72; IG: n=171  Type 2 diabetes | Non-personalised educational text message system | Tier B: Promoting good health | **Outcomes:** (1) At 12 months, compared with the control group (CG), the decrease of fasting plasma glucose (FPG) (1.5 vs. 0.4, *P*=.011) and control rate (49.4% vs. 33.3%, *P*=.034), the postprandial glucose (PPG) (5.8 vs. 4.2, *P*=.009) and control rate (57.8% vs. 33.7%, *P*=.002) were better in the intervention group (IG); (2) In terms of self-management, improvements in weight control (49.3% vs. 28.2%, *P*=.031), vegetables consumption (87.3% vs. 29.0%, *P*<.001), fruits consumption (27.5% vs. 7.4%, *P*=.022), and physical activities (84.7% vs. 70.0%, *P*=.036) were better in the IG than in the CG  **Enablers:** Participants had high adherence when health care professionals were able to use mobile phones to maintain close contact with participants to provide individualized education and management  **Barriers:** Self-management and patient's ability to follow recommendations can be limited by their environment and individual factors (e.g. availability of fresh food, food literacy, medical resources, education/knowledge about the concepts being promoted, lack of health promotion initiatives to complement individual interventions) |
| Yasmin et al., (2020) [36] | (1) To explore different factors (personal, familial, social, and financial) affecting both the life of patients with diabetes type 2 and the management of the disease; (2) To explore patient’s perception of a mobile health intervention in the context of disease management | Mixed-methods - sequential explanatory design | Bangladesh  Hospital  n = 320 patients (n= 160 IG, n = 160 CG) in RCT; n = 18 included in for qualitative study  Type 2 diabetes | mHealth comprising a patient reminder system to follow personalised recommendations and attend appointments, and a physician call centre | Tier B: Communicating about health and care | **Outcomes:** The cost of overall treatment and the lack of availability of safe public places for physical exercise and unfavourable weather conditions were mentioned as barriers to the overall management of diabetes  **Enablers:** Telephone calls can be used to better reach older people and people with low literacy and/or SES  **Policy/Practice:** (1) The availability of mHealth solutions on its own is not enough - healthcare infrastructure and the wider environment need to support patients to overcome barriers to self-management (e.g. lack of public exercise spaces); (2) A patient-centred mobile health intervention supported by a collaborative patient-provider relationship, a strong family support system, available public spaces for exercise and the introduction of a functional public health insurance system could be beneficial for the better management of diabetes |
| Zeng et al., (2020) [55] | To investigate the relationship between patient engagement in an mHealth intervention and depressive symptoms using repeated measures of both patient engagement and patient outcomes at 4 time points | Quantitative - secondary analysis of RCT data | China  HIV specialist hospital outpatients department  n = 150  People living with HIV who had elevated depressive symptoms | Run4Love WeChat-based intervention comprising an adapted cognitive-behavioural stress management (CBSM) course and personalised physical activities promotion | Tier C: Informing clinical management | **Outcomes:** Results of LGCMs revealed that two measures of patient engagement, completion rate and frequency of items completed, were significantly associated with reduced depressive symptoms, and these relationships occurred at 3 months of the intervention but not at 1 or 2 months. However, time spent on the program was not significantly related to depressive symptoms throughout the intervention |
| Zheng et al., (2019) [48] | To test whether a culturally sensitive text messaging intervention supporting secondary prevention improves the control of risk factors in patients with coronary heart disease in China | Quantitative - multicentre, single-blinded randomised controlled trial | China  Hospital  n = 822 patients  Coronary heart disease without diabetes | Text message system with educational and motivational material | Tier B: Promoting good health | **Outcomes:** (1) At 6 months, systolic blood pressure was not significantly lower in the intervention group compared with the control group, with a mean change (SD) of 3.2 (14.3) mmHg and 2.0 (15.0) mmHg (*P*>.05) from baseline, respectively (mean net change, −1.3 mmHg [95% CI, −3.3 to 0.8]; *P*=.221); (2) There were no significant differences in the change in LDL-C level, physical activity, body mass index, or smoking status between the 2 groups  **Policy/Practice:** Text messages providing educational and motivational information to advance secondary prevention goals were feasible to develop and were highly acceptable to participants |
